# Updates on BES1/BZR1 Regulatory Networks Coordinating Plant Growth and Stress Responses

**DOI:** 10.3389/fpls.2020.617162

**Published:** 2020-12-03

**Authors:** Alfredo Kono, Yanhai Yin

**Affiliations:** Department of Genetics, Development and Cell Biology, Plant Sciences Institute, Iowa State University, Ames, IA, United States

**Keywords:** brassinosteroid, BES1, BZR1, SUMOylation, jasmonic acid, auxin

## Abstract

Brassinosteroids (BRs) play pivotal roles in the regulation of many dimensions of a plant’s life. Hence, through extensive efforts from many research groups, BR signaling has emerged as one of the best-characterized plant signaling pathways. The key molecular players of BR signaling from the cell surface to the nucleus important for the regulation of plant growth and development are well-established. Recent data show that BRs also modulate plant responses to environmental stresses such as drought and pathogen infection. In this mini review, we present the recent progress in BR signaling specifically in the post-translational SUMO modification of BR’s master regulators, BES1/BZR1. We also discuss recent findings on the crosstalk between BR, UV light, and jasmonic acid signaling pathways to balance growth during light stress and pathogen infections. Finally, we describe the current update on the molecular link between BR signaling and intracellular auxin transport that essential for plant development.

## Introduction

Brassinosteroids (BRs) are plant steroid hormones that have a significant impact on plant growth and development, cell elongation, resistance to pathogens, and plant responses to environmental stresses. In the past decades, substantial progress in dissecting molecular mechanisms of the BR signaling pathway has been achieved using the model plant *Arabidopsis thaliana* (Nolan et al., 2020).

In *Arabidopsis*, BRs are perceived by a plasma membrane receptor, BRASSINOSTEROID INSENSITIVE1 (BRI1) leucine-rich repeat receptor-like kinase (LRR-RLK; Li and Chory, 1997; Wang et al., 2001; Kinoshita et al., 2005). BR binding to an extracellular LRR domain of BRI1 changes its conformation and allows the attachment of a required coreceptor, SOMATIC EMBRYOGENESIS RECEPTOR KINASE3 (SERK3)/BRI1-ASSOCIATED KINASE1 (BAK1; Li et al., 2002; Nam and Li, 2002; Hothorn et al., 2011; She et al., 2011; Santiago et al., 2013). The formation of BRI1-BAK1 complex triggers a cascade of intracellular phosphorylation events involving several regulatory proteins, which eventually inhibit the activity of GSK3-like kinase BRASSINOSTEROID INSENSITIVE2 (BIN2), a primary negative regulator of BR signaling pathway (Li et al., 2002). Inactivation of BIN2 initiates the accumulation of BRI1-EMS-SUPPRESSOR1 (BES1) and BRASSINAZOLE RESISTANT1 (BZR1), two key transcription factors in BR signaling (Wang et al., 2002; Yin et al., 2002), and promotes their nuclear localization. When BRs are low, BIN2 phosphorylates BES1 and BZR1, thus, prevents their nuclear localization, suppresses their DNA binding activity, and/or promotes their degradation (Nam and Li, 2002; Kim et al., 2009).

BES1 and BZR1 contain a basic helix-loop-helix (bHLH) like motif of DNA binding domain and are known as master regulators that modulate the expression of many target genes (He et al., 2005; Yin et al., 2005; Nolan et al., 2017a; Nosaki et al., 2018). These two transcription factors are subjected to various post-translational modifications that modulate their activity in response to environmental signals. They also have been identified as a node that connects BR signaling with other signaling pathways such as, light, auxin, and gibberellin (reviewed by Nolan et al., 2020). Here, we present updates on BR signaling with a focus on BES1 and BZR1 post-translational regulation and crosstalk between BR, jasmonic acid (JA), UV light, and auxin signaling pathways.

## Sumoylation of BES1 and BZR1

Regulation of BES1 and BZR1 occurs in multiple ways. Phosphorylation of BES1/BZR proteins is well studied, and additional mechanisms that involve oxidation, ubiquitination, alternative splicing, and degradation are emerging (reviewed by Nolan et al., 2020). Recently, post-translational modification of BZR1 and BES1 by small ubiquitin-like modifier (SUMO) reportedly alters their functionality. SUMOylation is a reversible modification in which SUMO protein is covalently conjugated to its substrate through a series of biochemical reactions similar to ubiquitination. In plants, SUMOylation influences many fundamental cellular processes, including environmental stress responses by governing target proteins stability, protein-protein interaction, and subcellular localization (Morrell and Sadanandom, 2019).

**Figure 1:**
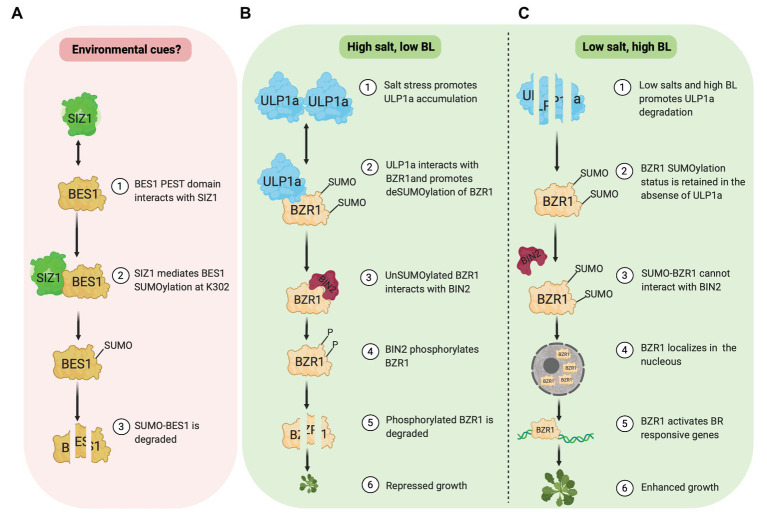
SUMOylation of BES1 (pink box) and BZR1 (green box). **(A)** E3 ligase, SIZ1, mediates SUMOylation of BES1 at K302, which promotes BES1 degradation. **(B)** Under salt stress, ULP1a SUMO protease catalyzes deSUMOylation of BZR1 and BIN2 phosphorylates non-SUMOylated BZR1, which leads to BZR1 degradation. **(C)** Under non-salt stress condition, SUMO-BZR1 is stable and localizes in the nucleus to activate BR responsive genes.

Zhang et al. (2019) reported that the SUMOylation of BES1 by E3 ligase SIZ1 destabilizes and inhibits BES1 activity (Figure 1A). SIZ1 and BES1 physically interact *in vivo* and *in vitro*. Amino acids 219–288 in BES1, which includes the PEST domain implicated in BES1 degradation, are required for its interaction with SIZ1. Furthermore, the BES1 SUMOylation site is identified at Lysine(K)302 although this SUMOylation status is unaffected by brassinolide (BL) application. Compared to wild-type seedlings, the accumulation of BES1 and unphosphorylated BES1 protein is higher in a SIZ1 T-DNA insertion line (*siz1-2*). In the presence of cycloheximide, a protein synthesis inhibitor, BES1 protein in wild type is less stable than in *siz1-2* seedlings with or without BR, suggesting a negative role of SIZ1 in BES1 stability. In agreement, a cell-free degradation assay shows that without a 26S proteasome inhibitor (MG132), MBP-BES1 protein degradation is slower when incubated with total protein extracts from *siz1-2* seedlings than that of the wild type. Meanwhile, MG132 presence prevents MBP-BES1 degradation in both WT and *siz1-2* extracts. Together, these data show that the SIZ1-mediated SUMOylation promotes BES1 instability and stimulates its proteasome-dependent degradation.

BES1 transcriptional activity is modulated by SIZ1-mediated SUMOylation (Zhang et al., 2019). Using a luciferase (LUC) reporter assay in *Arabidopsis* protoplast, they investigated the promoter activity of two BR repressed genes, *DWAF4* and AT2G210, and one BR induced gene, *SAUR-AC1*. As expected, wild-type BES1 represses the activity of *DWF4*‐ and AT2G4210-LUC. Meanwhile, BES1^K302R^ variant in which SIZ1 SUMOylation site is mutated, further represses the *DWF4-* and AT2G4210-LUC activity. Similarly, the activation level of *SAUR-AC1*-LUC activity is significantly higher in the presence of BES1^K302R^ variant rather than wild-type BES1. To further confirm this finding, the binding ability of BES1 to the promoter of *DWAF4*, AT2G210, and *SAUR-AC1* was investigated. Chromatin immunoprecipitation (ChIP) assay results showed that the BES1 binding ability increases in the *siz1-2* compared to the wild type regardless of MG132. Based on these data, Zhang et al. (2019) suggested that the SUMOylation of BES1 by SIZ1 inhibits its transcriptional activity.

Contrary to BES1, a new study by Srivastava et al. (2020) revealed that SUMOylation of BZR1 at different sites stabilizes its activity and promotes growth, specifically in non-stress conditions. Meanwhile, salinity (salt stress) provokes BZR1 deSUMOylation through a SUMO protease, ULP1a, which leads to BZR1 degradation and plant growth suppression (Figures 1B,C). Salt stress fails to suppress root growth in a *ulp1a* mutant compared to wild-type since *ulp1a* mutant seedlings have longer root than wild-type seedlings in the presence of sodium chloride. Interestingly, *ulp1a* mutant is more tolerant to BR biosynthesis inhibitor brassinazole (BRZ), suggesting that the BR signaling in the *ulp1a* mutant is upregulated. To confirm the *ulp1a* BR response, the expression of BR biosynthesis genes and BR activated genes were investigated. BR availability activates BR induced genes under normal BR signaling, while BR biosynthesis genes are suppressed to maintain BR homeostasis. Indeed, in the *ulp1a* mutant, the expression of BR biosynthesis genes and BR activated genes decreases and increases, respectively, confirming that the ULP1a is negatively impacting BR signaling (Srivastava et al., 2020).

*In silico* analysis of BR signaling components reveals that the BZR1 and its homologs in other plant species, including BES1 in *Arabidopsis*, contain two conserved lysine residues, K280 and K320, which likely serve as putative SUMO conjugation sites. Co-immunoprecipitation assays in *Nicotiana benthamiana* capture the physical interaction between BZR1 and ULP1a. Since ULP1a is a SUMO protease, this interaction suggests that the ULP1a may promote deconjugation of SUMO in BZR1. Indeed, subsequent *in vitro* and *in vivo* experiments in planta demonstrated that the ULP1a specifically mediates deSUMOylation of BZR1 (Srivastava et al., 2020). It would be interesting to see if ULP1a could also act on BES1 since both BES1 and BZR1 proteins are highly identical.

Upon BL treatment, ULP1a protein is unstable, and SUMOylated BZR1 protein increases. On the other hand, in the presence of BRZ, ULP1a protein accumulates, and promotes deSUMOylation of BZR1. Furthermore, dephosphorylation level of wild-type BZR1 increases after BL treatment. However, the level of phosphorylation and dephosphorylation of the BZR1 SUMO-deficient form remain constant, indicating the significant role of SUMOylation to the accumulation of BZR1 (Srivastava et al., 2020).

BZR1 and ULP1a co-localize in the cytoplasm, and SUMOylation of BZR1 modulates its nuclear distribution (Liao et al., 2020; Srivastava et al., 2020). While BL treatment increases nuclear-to-cytoplasmic (NP) ratio of wild-type BZR1, the NP ratio of BZR1 SUMO-deficient form is unchanged. In contrast, after BRZ treatment, the NP ratio of BZR1 SUMO-deficient form is rapidly decreased than that of the wild type. Interestingly, SUMOylation of BZR1 prevents its interaction with BIN2. The inability of BIN2 to interact with SUMO-BZR1 may be due to the overlap of SUMO site, K280, and K320, with BIN2 Interaction Motif, amino acids 309–320 (Peng et al., 2010), in BZR1. Since BIN2 is critical for phosphorylation which leads to BZR1 degradation, SUMOylation of BZR1 may play a significant role in maintaining BZR1 stability. DeSUMOylation of BZR1 by ULP1a is reportedly stimulated by salt stress (Srivastava et al., 2020). In this condition, ULP1a protein abundance is elevated, which results in decreasing SUMOylated BZR1 proteins. Thus, it is likely that the deSUMOylated form of BZR1 would be phosphorylated by BIN2 and degraded. This mechanism may be essential to control plant growth during salt stress (Figure 1B). A recent study showed that the BIN2 cooperates with calcium sensor SOS3/SCaBP8 to regulate SOS2 and BES1 to coordinate plant growth and growth recovery (Li et al., 2020b), suggesting that the BR and salt stress crosstalk at multiple targets.

## Crosstalk Between BR and Stress Signaling Pathways

BRs are involved in plant responses to biotic and abiotic stresses (Kohli et al., 2019; Gupta et al., 2020) and crosstalk between BRs and other plant hormones is well-documented (Bostock, 2005; Nolan et al., 2020). To synchronize plant growth and defense to pathogen infection, BRs and jasmonic acid (JA) signaling pathways functionally interact. In rice, the presence of BL promotes susceptibility to the brown planthopper (BPH) pathogen and induces root susceptibility to the root-knot nematode *Meloidogyne graminicola* (Nahar et al., 2013; Pan et al., 2018). The increased sensitivity to *M. graminicola* is associated with an antagonistic interaction between BR and JA pathways. Upon BL treatment, JA-related genes transcripts are downregulated in the root. Furthermore, BR deficient mutant shows elevated endogenous JAs and decreased susceptibility to *M. graminicola* (Nahar et al., 2013). In contrast, upon BPH infestation, BR stimulates JA signaling while represses salicylic signaling pathway (Pan et al., 2018), suggesting a different mechanism of plant immunity responses.

**Figure 2:**
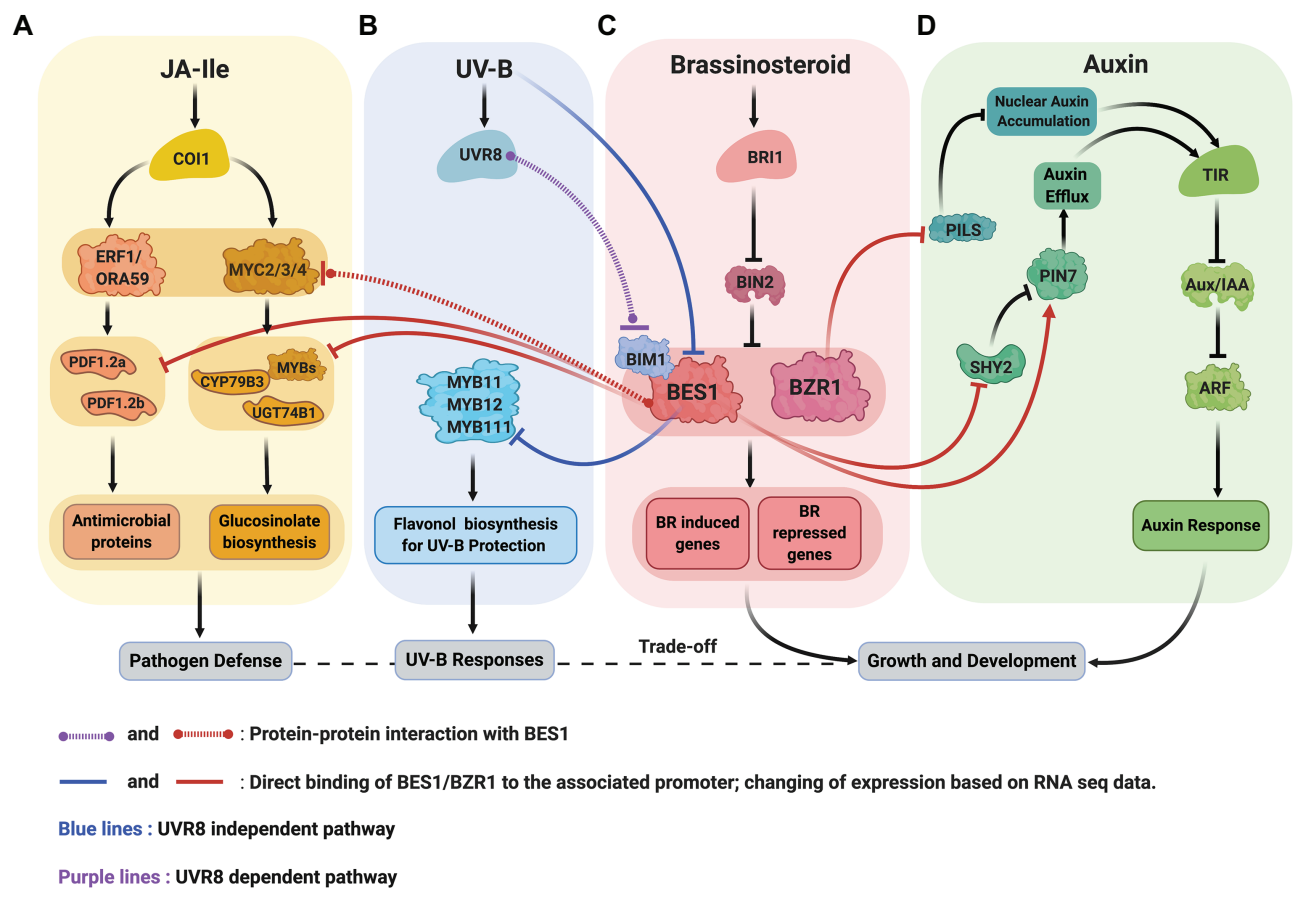
Crosstalk between signaling pathway of BR (**C**, red), JA (**A**, yellow), UV-B (**B**, blue), and auxin (**D**, green). **(A,C)** BR and JA: BES1 restricts the production of defensin (antimicrobial proteins) by repressing the expression defensin genes, PDF1.2a and PDF1.2b. BES1 also negatively regulates the production of glucosinolates (GS) by inhibiting the transcriptional activity of MYC2, repressing GS biosynthesis transcription factors (MYBs) and genes (CYP79B3 and UGT74B1). **(B,C)** BR and UV-B: UV-B activated UVR8 and represses the function of BES1-BIM1 complexes to reduce the expression of BR induced genes to balance growth and stress (UVR8 dependent pathway). UV-B also reduces BES1 expression allowing the expression of MYB11, MYB12, and MYB111 transcription factors critical for flavonol biosynthesis (UVR8 independent pathway). **(C,D)** BR and auxin: BZR1 reduces the abundance of PILS protein (ER localized) resulting in auxin’s nuclear accumulation. BES1 increases the abundance of PIN7 that involved in internal auxin flow in the embryo.

Recent data from *Arabidopsis* revealed the molecular mechanisms underlying BR-JA pathway crosstalk that mediate the growth-defense trade-off (Figures 2A,C). Liao et al. (2020) discovered that BRs antagonize JA-activated defense mechanism *via* BES1, a master regulator in BR signaling pathway. Two routes in which BES1 represses JA-activated defense pathway were established. First, BES1 represses the expression of genes involved in the production of small cysteine-rich peptides with antimicrobial activity known as defensins (Thomma et al., 2002). Second, BES1 represses glucosinolate (GS) biosynthesis genes that produces GS compounds crucial for plant resistance to insect (Hopkins et al., 2009). In *bes1-D* mutant, defensin genes such as *PDF1.2a* and *PDF1.2b*, also a JA marker gene, *VPS1*, are downregulated. Meanwhile, in *bzr1-1D* mutant, *PDF1.2b*, and *VPS1* transcripts are upregulated, suggesting that the BES1 has more dominant role in the suppression of JA-activated defense genes. A chromatin immunoprecipitation (ChIP)-qPCR, an electrophoretic mobility shift assay (EMSA), and a dual-luciferase (LUC) reporter assay confirmed that the BES1 directly binds the 3' downstream region of *PDF1.2a* and *PDF1.2b* and suppresses their expression.

Mutant of *bes1-D* is more susceptible to *Botrytis cinerea* infection than wild-type, while stable overexpression lines of *PDF1.2a* show improved resistance to *B. cinerea*. In response to *B. cinerea* invasion, the double mutant, *bes1-D* and *PDF1.2a* overexpression lines, is more similar to *PDF1.2a* overexpression lines than *bes1-D* (Liao et al., 2020). These genetic data support the antagonistic role of BES1 in JA mediated defense response.

The *bes1-D* mutant shows increased sensitivity to *Spodoptera exigua* (Liao et al., 2020). Furthermore, the level of glycosynolates (GS), which functions downstream of JA, significantly decreases in *bes1-D* mutant as well as the expression of indolic GS synthesis genes. BES1 modulates GS biosynthesis in two ways. First, the ChIP-qPCR and LUC reporter assay results demonstrated that the BES1 directly binds and represses the expression of indolic GS-biosynthetic genes, *cytochrome P450 family79 subfamily B polypeptide3 (CYP79B3)* and *UDP-glucosyl transferase 74B1 (UGT74B1)*, as well as the expression of *MYB34*, *MYB51*, and *MYB122* transcription factors that specifically regulate the expression indole-GS genes. Second, BES1 suppresses the transcriptional activity of MYC2 that involved in the activation of GS-biosynthetic genes. In the LUC reporter assay, the co-expression of both BES1 and MYC2 further represses the activity of *CYP79B3-LUC* and *UGT74B1-LUC* activity compared to MYC2 expression alone (Liao et al., 2020).

Consistent with the general theme that BES1/BZR1 functions as a hub to coordinate growth and stress responses, recent studies showed that the BES1 mediates UV-B stress responses at multiple levels (Figures 2B,C). First, UV-B receptor UVB8 represses the functions of BES1 and BIM1 and thus inhibit BR-induced plant growth (Liang et al., 2018). Secondly, the expression of *MYB11/12/111* genes required for flavonol biosynthesis is negatively regulated by BES1 under normal light condition and broad band UV-B reduces BES1 expression, allowing the production of flavonol for the UV-B stress protection (Liang et al., 2020).

## Crosstalk Between BR and Auxin Signaling Pathways

Crosstalk between BRs and auxin are widely known, and the molecular link underlying this relationship has been reported. For example, the interplay between auxin transcriptional regulators, the auxin response factor (ARF) family, and BR signaling components, such as BIN2 alters the expression of auxin-responsive genes. Phosphorylation of ARF2 eliminates its DNA binding and repressor activity. Furthermore, around 40% of differentially expressed genes in *arf2* loss-of-function mutant also are responsive to BRZ (Vert et al., 2008). Recently, BR signaling reportedly modulates intracellular auxin transport *via* auxin transporter proteins, PIN-FORMED (PIN), and PIN-LIKES (PILS) family proteins (Li et al., 2020a; Sun et al., 2020; Figures 2C,D).

Li et al. (2020a) showed that the interaction between BR signaling and auxin promotes root meristem development. BR-deficient mutants expressing auxin DR5::GFP marker exhibit lower fluorescence intensity in the seedling stage and reduced root meristem size. Upon exogenous auxin application, root meristem size, length, and cell number partially recovered. Furthermore, they showed that the BR elevates the expression level of PIN7. The PIN proteins are involved in cell-to-cell transport and intracellular auxin accumulation, which is essential for the root gravitropic response and aboveground organogenesis (Adamowski and Friml, 2015). PIN7, along with PIN1, PIN3, PIN4, and PIN5 are expressed in the embryo and determined the polarity of the embryonic axis (Friml et al., 2003). The upregulation of PIN7 in the BR treatment is mediated by BES1 that binds directly to the promoter of PIN7. BES1 also appears to regulate the expression of SHY2, a transcriptional repressor that inhibits the expression of PIN1, PIN3, and PIN7 (Ioio et al., 2008; Li et al., 2020a). EMSA and ChIP studies demonstrated that the SHY2 promoter is a direct target of BES1. BES1 participation in transcriptional regulation of SHY2 was shown by monitoring GUS expression driven by SHY2 promoter in BES1-RNAi and wild-type seedlings. The results indicated that the intensity of GUS signals is higher in BES1-RNAi mutants compared to wild-type. In line with this data, SHY2 transcript levels are significantly higher in BES1-RNAi lines than wild type. These results are consistent with the notion that BES1 negatively regulates SHY2. The connection between SHY2 and PINs was investigated by examining the expression of PIN1-GFP, PIN3-GFP, and PIN7-GFP driven by their native promoter in a loss-of-function mutant, *shy2-31*, and a wild-type background. The fluorescence signals of PIN1, PIN3, and PIN7 are higher in the *shy2-31* than wild type, which argue that the SHY2 inhibits the expression of those PIN proteins. Moreover, in the *shy2-31* background, BL further elevates fluorescence signals of PIN1, PIN3, and PIN7 while BRZ reduces the signals. Thus, Li et al. (2020a) conclude that in addition to direct regulation of *PIN7* by BES1, the expression of some of *PIN* genes including *PIN7* is partially regulated by BRs *via* BES1 and SHY2.

BR also regulates PILS-mediated intracellular auxin transport. PILS proteins evolve independently from PIN proteins, although they are structurally similar (Feraru et al., 2012). The role of PILS proteins in the developmental processes is mostly unknown. However, PILS proteins reportedly restrict nuclear auxin accumulation presumably by auxin retention in the ER resulted in spatially defined auxin minima that control organ growth responses (Feraru et al., 2019). PILS also integrates external signals such as light and temperature to the auxin signaling pathway (Sauer and Kleine-Vehn, 2019).

The connection between BR and PILS was revealed through forward-genetic screening in a PILS5 overexpressing strain (*PILS5^OE^*) to identify secondary mutations with enhanced hypocotyl and root growth deficiency (Sun et al., 2020). The identified mutant, *imperial pils (imp)*, carries a secondary mutation that surprisingly mapped to the BRI1 gene. The identified mutation creates a single amino acid change, glycine to serine, and the impact of this mutation resembles that of the previously identified weak loss-of-function *bri1* mutant, *bri1-6*, or *bri1-119* (Sun et al., 2020).

BR signaling appears to suppress PILS transcript and protein levels. *In silico* analysis showed that the promoters PILS2, PILS3, and PILS5 are direct targets of BZR1 and BES1 (BZR2) and the binding of BZR1 to PILS2 and PILS5 promoter was confirmed by chromatin immunoprecipitation (ChIP)-sequencing (Sun et al., 2020). Upon BL application, the expression of GFP and GUS fused to the promoter of PILS2, PILS3, and PILS5 is repressed as well as the transcript level of endogenous PILS3 and PILS5. Similarly, pPILS5::GFP-GUS signals in roots increase in *bri1* loss-of-function mutants but decrease in BRI1 overexpressing lines and in the constitutive mutant, *bzr1-1D*. The later supports the notion that the BZR1 transcription factor may negatively regulate PILS5 expression. PILS5-GFP signals driven by 35S promoter declines upon BL treatment. In contrast, the same construct signals are increased in a 3-day-old seedling of *bri1^imp^* (a *bri1* allele isolated from forward-genetic screening in *PILS5^OE^*) than the wild type, suggesting a non-transcriptional regulation of PILS5 expression by BR signaling (Sun et al., 2020).

BR directly modulates PILS activity in intracellular auxin relocation, which subsequently contributes to the regulation of root organs. After BL treatment, nuclear auxin abundance is increased; meanwhile, PIL3 and PIL5 protein abundance are decreased, and PIL6 protein abundance, on the other hand, is only slightly reduced. In contrast, BL induction in a PIL6 constitutive line partially limits nuclear auxin accumulation. Furthermore, the triple mutant of PILS shows accelerated auxin signaling and hypersensitivity to BL application in the root. These data suggest that BRs increase nuclear auxin accumulation in the root by decreasing the presence of PILS proteins (Sun et al., 2020).

## Conclusions

In harsh environmental conditions, plants cannot move around; thus, fine-tuning their growth and development using sophisticated internal signaling networks enable plants to survive and to conclude their life cycle. BR signaling is essential in regulating plant growth in response to environmental stresses. For example, *Arabidopsis* coordinates its growth and survival under drought *via* RD26 transcription factor that binds and antagonizes BES1 transcriptional activities to inhibit BR-mediated growth (Ye et al., 2017). BES1 also interacts with WRKY54 and AP2/ERF TINY transcription factors to balance growth and drought response (Chen et al., 2017; Xie et al., 2019). Furthermore, activation of ubiquitin receptor protein DSK2 in drought prompts BES1 degradation through autophagy (Nolan et al., 2017b). New studies by Srivastava et al. (2020) and Sun et al. (2020) indicate that SUMO modifications of BZR proteins are essential not only in modulating the activity of these transcription factors but also crucial in adjusting plant growth under salt stress, as seen in the case of BZR1. It is quite intriguing that the SUMOylation of BES1 and BZR1 produces opposite outcomes, where BES is degraded while BZR1 is more stable. The different results could be due to the distinct SUMO target sites in each protein. Furthermore, it needs to be confirmed if SIZ1 and ULP1a could act on both BZR1 and BES1. While ULP1a mediated deSUMOylation strongly correlated with salt stress, it is still unclear if specific environmental cues dictate the SUMOylation of BES1 and BZR1. Recent studies also indicated that the light and nitrogen starvation can affect BZR1 phosphorylation status. In *Arabidopsis*, cryptochrome 1 (CRY1), a blue light receptor involved in the inhibition of hypocotyl elongation, interacts with BZR1 and suppresses BZR1 DNA binding ability. CRY1 also promotes the BZR1 phosphorylation to prevent its nuclear localization (He et al., 2019). Meanwhile, in tomato, to alleviate nitrogen deficiency stress, BR induces autophagy through BZR1 that directly binds to the promoter of ATG2 and ATG6 to increase their expression and promotes autophagosome formation. During nitrogen starvation, BZR1 was dephosphorylated especially in the first 5 days of nitrogen starvation (Wang et al., 2019). Investigating how different modifications collectively modify BES1/BZR1 in response to different environmental cues will be an important future area of research.

Molecular mechanism behind the antagonistic interaction between BR and JA signaling in response to pathogen attacks gives some clues on how plants balance their growth in this condition. It appears that the BES1 is emerging as a node that connects BR and JA signaling and it negatively modulates different aspect of JA induced plant defense depending on the type of pathogens. In response to *B. cinereal* infection, BES1 directly suppresses the expression of defensins genes, PDF1.2a and PDF1.2b. On the other hand, upon exposure to *S. exigua* herbivory infection, BES1 negatively modulates MYC2 transcriptional activities. BES1 also represses the expression of indol-GS synthetic genes, *CYP79B3* and *UGT74B1* and GS-associated transcription factors. This negative regulation mediated by BES1 may be crucial to maintain homeostasis of defensins and GS compounds that are increasing upon pathogen attacks (Liao et al., 2020).

Additional layers of hormone crosstalk have been depicted by how BR signaling modulates the intracellular auxin distribution. BR signaling is directly repressing the expression of PILS proteins that restrict nuclear auxin availability (Sun et al., 2020). BR signaling also modulates the expression of PIN7, which participates in polar auxin transport in the root meristem (Li et al., 2020a). However, the mechanistic basis of how specific BR signaling components influence PILS protein stability remains to be established. Nonetheless, these data reveal new insights on how plants synchronize BR and auxin signaling to support growth and development.

## Author Contributions

AK and YY conceived the idea and wrote the review. All authors contributed to the article and approved the submitted version.

### Conflict of Interest

The authors declare that the research was conducted in the absence of any commercial or financial relationships that could be construed as a potential conflict of interest.
